# Charnley low-friction arthroplasty of the hip. Five to 25 years survivorship in a general hospital

**DOI:** 10.1186/1471-2474-9-69

**Published:** 2008-05-15

**Authors:** Daniel Hernández-Vaquero, Abelardo Suárez-Vazquez, Jesus Fernandez-Lombardia

**Affiliations:** 1 Department of Orthopaedic Surgery, Hospital St Agustin, Aviles, Spain; 2 School of Medicine, University of Oviedo, Spain

## Abstract

**Background:**

Some studies have raised the question about whether the good results obtained with the Charnley prosthesis could be replicated at general hospitals when it comes to the frequency of early complications and failure rates, both of which would be higher than those published by centres devoted to hip arthroplasties.

**Methods:**

We reviewed the results of 404 Low Friction Arthroplasties of the hip implanted between 1976 and 1993 in a general hospital by general orthopaedic surgeons. For the survival analysis, the end-point chosen would be the chirurgical revision of any of the prosthetic components for whatever reason.

**Results:**

The complications were 16 dislocations (4%), 14 deep infections (3.5%), 2 neurological injuries (0,5%) and 5 clinical deep venous thromboses (1.2%) (2 pulmonary embolisms). The survival rate at 25 years, both for stem and cup, was 83%. Survival was higher in those arthroplasties implanted in patients older than 60 years, with statistical significance.

**Conclusion:**

Low Friction Arthroplasty undertaken at general hospitals by general orthopaedic surgeons feature similar outcomes to those found in centres devoted to hip surgery.

## Background

Low Friction Arthroplasty (LFA) is still considered the "golden guide" when it comes to compare the different Total Hip Arthroplasy (THA) models. Many studies have been published on LFA, showing excellent, long-term results, both about clinical and radiological issues [1,2] as well as about survival analyses [3-5]. Nevertheless, most of these studies include series of LFA undertaken at hospitals which were pioneers in this technique [6], or which were devoted to hip surgery procedures. Some studies have raised the question about whether the good results obtained with the Charnley prosthesis could be replicated at general hospitals when it comes to the frequency of early complications and failure rates, both of which would be higher than those published [7]. Survival analysis is a powerful tool for analysing the results of total joint replacements despite the objections found in the literature [8], but it has major drawbacks when the failure rates are very low.

**Figure 1:**
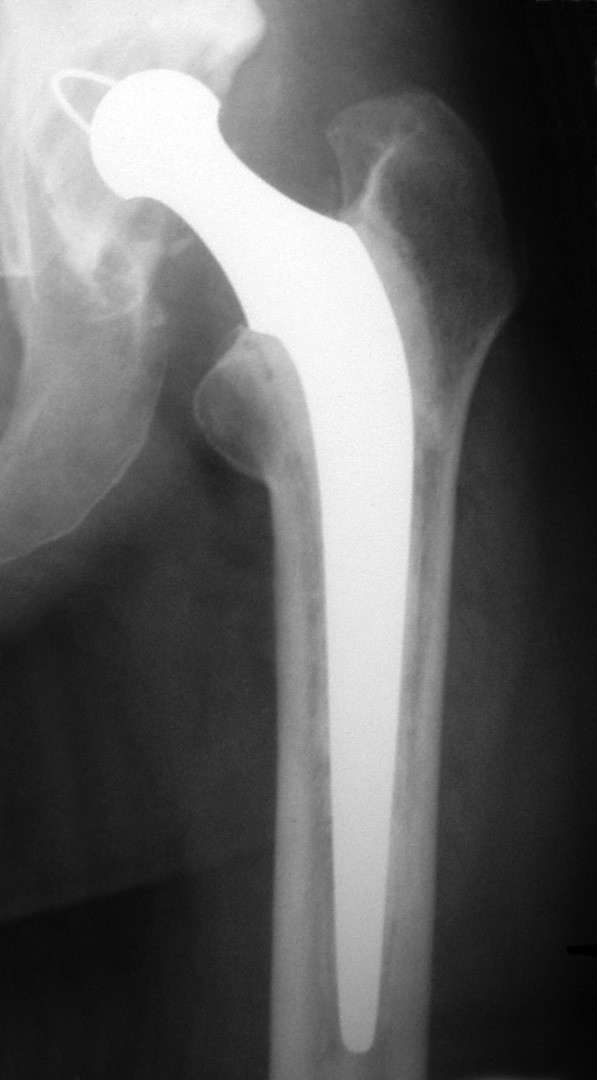
**Low friction arthroplasty with 22 years follow-up.** No complications.

Our Unit belongs to a general hospital located in northern Spain, and it serves a population of 180,000; it is not specifically devoted to hip surgery. Since its opening in 1976, the hospital has remained faithful to LFA throughout the years (Figure 1). Our aim is to know the long term behaviour of these arthroplasties by studying any eventual complications which may have arisen and by conducting a survival analysis.

## Methods

404 LFA (294 patients) performed as primary procedures consecutively from 1976 to 1993 have been included in this study. In all cases the implant used was the prosthesis designed by J. Charnley, with the technical and design improvements added throughout the years [9]. The surgical procedures were undertaken by a team of six general orthopaedic surgeons following a homogeneous technique, always using a Smith-Petersen anterior approach without performing the osteotomy of the greater trochanter. The senior surgeon (DHV) had previously attended to the Center for Hip Surgery at Wrightington, England in 1976 and 1979 to learn the original technique. No mechanical cementing system was used.

Throughout 2004, two independent observers who did not participate in the surgery (ASV and JFL) reviewed the medical histories and radiographies of each patient, categorizing the radiographs into those that were possibly, probably or definitely loose. General data about the patient and the intervention as well as about the clinical development were gathered. Since we are dealing with a long-term, retrospective study, only easily verifiable variables (age, sex, side) were accepted. Any complications which required an active medical intervention and which, accordingly, were featured in the medical histories, were also included.

For the survival analysis, the end-point chosen would be the chirurgical revision of any of the prosthetic components for whatever reason. Variable data (time, event, age and sex) were recorded in a SIGMASTAT v 3.00 for Windows (SPSS, Inc., Chicago, IL) database built for such purpose. The survival calculations were done with this tool, following the Kaplan-Meier method [10] with a confidence interval of 95%. The equality of survival distributions was contrasted for the acetabulum and stem components through the Log Rank test. In every hypothesis test, only p < 0.05 values were considered statistically significant.

## Results

The mean age at the moment of surgery was of 67 years (SD: 8.8), where the maximum age was 91 years and the minimum was 36. 57% of the cases were males and 43% females; 45% were right side and 55%, left side.

The following complications were registered: 16 dislocations (4%), 14 deep infections (3.5%), 5 periprosthetic fractures (1.2%), 2 neurological injuries (in the crural nerve and 1 in the sciatic nerve), 5 deep venous thromboses (1.2%) with 2 pulmonary embolisms, one of them fatal.

**Figure 2:**
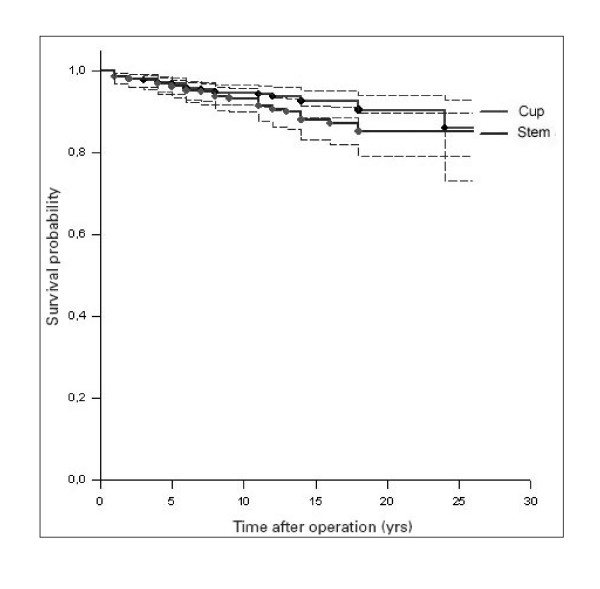
Kaplan-Meier survival curve showing the cumulative survival per year.

**Figure 3:**
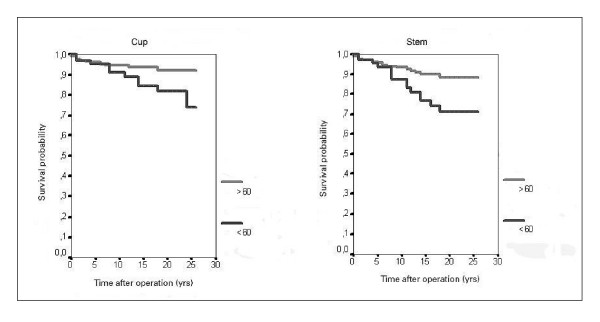
Survival curve of the components after grouping the series according to the age of the patients.

After 25 years, the cup and stem survival rates were of 83% (Table 1 and 2), (Figure 2). A 55.2% of arthroplasties were lost by dead of patients and 20% by unknown causes [see Additional file 1]. The acetabular component demonstrated a better behaviour at the beginning of the follow-up, but at the end it equals that of the femoral stem (though it has no statistical significance). Figure 3 shows the survival curve of the components after grouping the series according to the age of the patients (younger and older than 60 years). Survival was higher in those arthroplasties implanted in patients older than 60 years, with statistical significance in the Log Rank test, both for the acetabular component (p = 0.008) and the femoral stem component (p = 0.043), with a higher difference between the curves as development time increases. There were no differences related to sex.

**Table 1:** Survival rate of the cup

Follow-up (years)	LFA	Cup survival (%)
5	307	96
10	235	93
15	128	91
20	57	89
25	10	83

**Table 2:** Survival rate of the stem

Follow-up (years)	LFA	Stem survival (%)
5	305	95
10	230	92
15	123	87
20	52	83
25	10	83

## Discussion

A surgical technique is reproducible if its results are similar in real, non-ideal, conditions, and that is what seems to happen with LFA. In the long term, the survival rate of LFA in our centre is very similar to those shown in historical series or arthroplasty register [11,12] and in those centres devoted to hip surgery [3,4,13] The worst survival rate is found in the youngest patients, just as these authors proved [12]. In the short-term, however, our survival rate is worse than those found in the aforementioned series, being closer to the results expressed by Fender et al [7]. Our deep infection rate is high, since it includes procedures undertaken in a time when antibiotic prophylaxis was not systematically used and this may justify, if only partially, such results.

Both in our series as well as in a multicentric series [14], the global survival rate was of 83% after 20 years. There are, however, some minor differences when we analyze the survival of each component on its own, around 87% for each of them in Older's multicentric series, and 83% and 89% for the acetabular and stems, respectively, in our series.

The dislocation rate is also higher than the classical rate of 2%–3% for primary surgery [15] being closer to 3.9%, which can be found in those studies undertaken at general facilities [16]. However, it is still inferior to that published by Fender et al which reached 5%.

The assessment of results in THA requires a multiple approach, either through clinical score sets complemented with imaging analyses, curves or survival analysis, or through score and review indices about the quality of life related to health. Each approach addresses a requirement of the complex evaluation of this technique which, paradoxically enough, must be reviewed in the long term in spite of being relatively new and rapidly evolving in developed countries.

**Figure 4:**
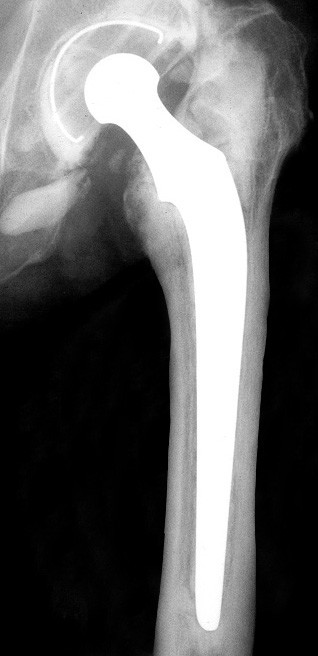
Stem loosening in a Charnley arthroplasty (17 years follow-up).

There remain many unresolved questions in hip replacement. Well-constructed clinical tests offer the best hopes of answering them. Such tests are onerous, and time and money are required to collect, record and analyze the data. The relatively few clinicians who undertake such tests must have an understanding of the statistical methods used and the problems they may encounter. It is especially difficult to find series where you can compare long term results in the literature and whether the arthroplasty was implanted in general hospitals. Only retrospective studies are available, which must be accepted in spite of the known issues with validity. The methodological flaws common to these studies could be alleviated using, as in our case, data about which there is absolute certainty they are true, even though this would mean an obvious loss of information. In joint replacement surgery the usual end-point is the decision to remove the prosthesis and revise or convert it to some other form of treatment. This end-point has been criticized because the criteria used to decide the need for removal will differ between patients and surgeons. This form of analysis defines a failure point or terminal event and provides an assessment of not only how many failures there had been but also how long after the operation did the failure occur. It does not measure function or pain, unless they are included in the definition of failure (Figure 4). Using a confidence interval of 95% in the survival curve allows a more faithful comparison of the best and worst possible results with those of other series.

To sum up, our study seems to confirm a higher frequency of early complications and a higher assessment rate of the THA in general hospitals compared to those in dedicated centres, although in the long term this trend does not keep up and the survival rates become similar. LFA undertaken at general hospitals by general orthopaedic surgeons feature similar outcomes to those found in centres devoted to hip surgery. A national joint replacement registry would be a useful tool to compare outcomes for high and low volume community hospitals as well as academic centres.

## Conclusion

Low Friction Arthroplasty undertaken at general hospitals by general orthopaedic surgeons feature similar outcomes to those found in centres devoted to hip surgery.

## Abbreviations

THA: Total hip arthroplasty; LFA: Low friction arthroplasty.

## Competing interests

The authors declare that they have no competing interests.

## Authors' contributions

DHV conceived of the study, participated in its design and coordination and drafted the manuscript, ASV and JFL reviewed the medical histories and radiographies. All authors read and approved the final manuscript.

## Pre-publication history

The pre-publication history for this paper can be accessed here:



## Supplementary Material

Additional file 1 Survival tables. 3 tables. Click here for file
