# Supplementary material for: Charnley low-friction arthroplasty of the hip. Five to 25 years survivorship in a general hospital
Source: BMC Musculoskelet Disord. 2008 May 15;9:69. doi: 10.1186/1471-2474-9-69 (PMC2426685; doi:10.1186/1471-2474-9-69)
Supplement: Additional file 1 — Survival tables. 3 tables. [file 1471-2474-9-69-S1.doc]

Cup survival (years). Life Table Survival Variable AC

Number Number Number Number Cumul SE of SE of

Intrvl Entrng Wdrawn Exposd of Propn Propn Propn Proba- Cumul Proba- SE of

Start this During to Termnl Termi- Sur- Surv bility Hazard Sur- bility Hazard

Time Intrvl Intrvl Risk Events nating viving at End Densty Rate viving Densty Rate

------ ------ ------ ------ ------ ------ ------ ------ ------ ------ ------ ------ ------

,0 404,0 9,0 399,5 3,0 ,0075 ,9925 ,9925 ,0075 ,0075 ,0043 ,0043 ,0044

1,0 392,0 37,0 373,5 6,0 ,0161 ,9839 ,9765 ,0159 ,0162 ,0077 ,0065 ,0066

2,0 349,0 22,0 338,0 2,0 ,0059 ,9941 ,9708 ,0058 ,0059 ,0087 ,0041 ,0042

3,0 325,0 11,0 319,5 1,0 ,0031 ,9969 ,9677 ,0030 ,0031 ,0092 ,0030 ,0031

4,0 313,0 4,0 311,0 2,0 ,0064 ,9936 ,9615 ,0062 ,0065 ,0101 ,0044 ,0046

5,0 307,0 17,0 298,5 1,0 ,0034 ,9966 ,9583 ,0032 ,0034 ,0106 ,0032 ,0034

6,0 289,0 13,0 282,5 3,0 ,0106 ,9894 ,9481 ,0102 ,0107 ,0120 ,0058 ,0062

7,0 273,0 11,0 267,5 1,0 ,0037 ,9963 ,9446 ,0035 ,0037 ,0125 ,0035 ,0037

8,0 261,0 15,0 253,5 2,0 ,0079 ,9921 ,9371 ,0075 ,0079 ,0134 ,0052 ,0056

9,0 244,0 9,0 239,5 ,0 ,0000 1,0000 ,9371 ,0000 ,0000 ,0134 ,0000 ,0000

10,0 235,0 20,0 225,0 ,0 ,0000 1,0000 ,9371 ,0000 ,0000 ,0134 ,0000 ,0000

11,0 215,0 22,0 204,0 1,0 ,0049 ,9951 ,9325 ,0046 ,0049 ,0141 ,0046 ,0049

12,0 192,0 29,0 177,5 1,0 ,0056 ,9944 ,9273 ,0053 ,0056 ,0150 ,0052 ,0056

13,0 162,0 18,0 153,0 ,0 ,0000 1,0000 ,9273 ,0000 ,0000 ,0150 ,0000 ,0000

14,0 144,0 14,0 137,0 2,0 ,0146 ,9854 ,9137 ,0135 ,0147 ,0176 ,0095 ,0104

15,0 128,0 14,0 121,0 ,0 ,0000 1,0000 ,9137 ,0000 ,0000 ,0176 ,0000 ,0000

16,0 114,0 17,0 105,5 ,0 ,0000 1,0000 ,9137 ,0000 ,0000 ,0176 ,0000 ,0000

17,0 97,0 6,0 94,0 ,0 ,0000 1,0000 ,9137 ,0000 ,0000 ,0176 ,0000 ,0000

18,0 91,0 19,0 81,5 2,0 ,0245 ,9755 ,8913 ,0224 ,0248 ,0232 ,0157 ,0176

19,0 70,0 13,0 63,5 ,0 ,0000 1,0000 ,8913 ,0000 ,0000 ,0232 ,0000 ,0000

20,0 57,0 10,0 52,0 ,0 ,0000 1,0000 ,8913 ,0000 ,0000 ,0232 ,0000 ,0000

21,0 47,0 9,0 42,5 ,0 ,0000 1,0000 ,8913 ,0000 ,0000 ,0232 ,0000 ,0000

22,0 38,0 6,0 35,0 ,0 ,0000 1,0000 ,8913 ,0000 ,0000 ,0232 ,0000 ,0000

23,0 32,0 12,0 26,0 ,0 ,0000 1,0000 ,8913 ,0000 ,0000 ,0232 ,0000 ,0000

24,0 20,0 9,0 15,5 1,0 ,0645 ,9355 ,8338 ,0575 ,0667 ,0597 ,0556 ,0666

25,0 10,0 5,0 7,5 ,0 ,0000 1,0000 ,8338 ,0000 ,0000 ,0597 ,0000 ,0000

26,0+ 5,0 5,0 2,5 ,0 ,0000 1,0000 ,8338 ** ** ,0597 ** **

** These calculations for the last interval are meaningless.

This subfile contains: 404 observations

The median survival time for these data is 26,00+

Stem survival (years). Life Table Survival Variable AV

Number Number Number Number Cumul SE of SE of

Intrvl Entrng Wdrawn Exposd of Propn Propn Propn Proba- Cumul Proba- SE of

Start this During to Termnl Termi- Sur- Surv bility Hazard Sur- bility Hazard

Time Intrvl Intrvl Risk Events nating viving at End Densty Rate viving Densty Rate

------ ------ ------ ------ ------ ------ ------ ------ ------ ------ ------ ------ ------

,0 404,0 9,0 399,5 4,0 ,0100 ,9900 ,9900 ,0100 ,0101 ,0050 ,0050 ,0050

1,0 391,0 37,0 372,5 6,0 ,0161 ,9839 ,9740 ,0159 ,0162 ,0081 ,0065 ,0066

2,0 348,0 22,0 337,0 2,0 ,0059 ,9941 ,9683 ,0058 ,0060 ,0090 ,0041 ,0042

3,0 324,0 11,0 318,5 ,0 ,0000 1,0000 ,9683 ,0000 ,0000 ,0090 ,0000 ,0000

4,0 313,0 4,0 311,0 4,0 ,0129 ,9871 ,9558 ,0125 ,0129 ,0109 ,0062 ,0065

5,0 305,0 17,0 296,5 2,0 ,0067 ,9933 ,9494 ,0064 ,0068 ,0117 ,0045 ,0048

6,0 286,0 13,0 279,5 3,0 ,0107 ,9893 ,9392 ,0102 ,0108 ,0130 ,0059 ,0062

7,0 270,0 11,0 264,5 1,0 ,0038 ,9962 ,9356 ,0036 ,0038 ,0134 ,0035 ,0038

8,0 258,0 15,0 250,5 3,0 ,0120 ,9880 ,9244 ,0112 ,0120 ,0147 ,0064 ,0070

9,0 240,0 9,0 235,5 1,0 ,0042 ,9958 ,9205 ,0039 ,0043 ,0152 ,0039 ,0043

10,0 230,0 19,0 220,5 ,0 ,0000 1,0000 ,9205 ,0000 ,0000 ,0152 ,0000 ,0000

11,0 211,0 19,0 201,5 4,0 ,0199 ,9801 ,9022 ,0183 ,0201 ,0174 ,0091 ,0100

12,0 188,0 29,0 173,5 2,0 ,0115 ,9885 ,8918 ,0104 ,0116 ,0187 ,0073 ,0082

13,0 157,0 18,0 148,0 1,0 ,0068 ,9932 ,8858 ,0060 ,0068 ,0195 ,0060 ,0068

14,0 138,0 12,0 132,0 3,0 ,0227 ,9773 ,8657 ,0201 ,0230 ,0223 ,0115 ,0133

15,0 123,0 13,0 116,5 ,0 ,0000 1,0000 ,8657 ,0000 ,0000 ,0223 ,0000 ,0000

16,0 110,0 18,0 101,0 1,0 ,0099 ,9901 ,8571 ,0086 ,0100 ,0236 ,0085 ,0100

17,0 91,0 6,0 88,0 ,0 ,0000 1,0000 ,8571 ,0000 ,0000 ,0236 ,0000 ,0000

18,0 85,0 18,0 76,0 2,0 ,0263 ,9737 ,8345 ,0226 ,0267 ,0279 ,0157 ,0189

19,0 65,0 13,0 58,5 ,0 ,0000 1,0000 ,8345 ,0000 ,0000 ,0279 ,0000 ,0000

20,0 52,0 9,0 47,5 ,0 ,0000 1,0000 ,8345 ,0000 ,0000 ,0279 ,0000 ,0000

21,0 43,0 7,0 39,5 ,0 ,0000 1,0000 ,8345 ,0000 ,0000 ,0279 ,0000 ,0000

22,0 36,0 5,0 33,5 ,0 ,0000 1,0000 ,8345 ,0000 ,0000 ,0279 ,0000 ,0000

23,0 31,0 12,0 25,0 ,0 ,0000 1,0000 ,8345 ,0000 ,0000 ,0279 ,0000 ,0000

24,0 19,0 9,0 14,5 ,0 ,0000 1,0000 ,8345 ,0000 ,0000 ,0279 ,0000 ,0000

25,0 10,0 5,0 7,5 ,0 ,0000 1,0000 ,8345 ,0000 ,0000 ,0279 ,0000 ,0000

26,0+ 5,0 5,0 2,5 ,0 ,0000 1,0000 ,8345 ** ** ,0279 ** **

** These calculations for the last interval are meaningless.

This subfile contains: 404 observations

The median survival time for these data is 26,00+

# Cup or Stem survival (years). Life Table Survival Variable ACOV

Number Number Number Number Cumul SE of SE of

Intrvl Entrng Wdrawn Exposd of Propn Propn Propn Proba- Cumul Proba- SE of

Start this During to Termnl Termi- Sur- Surv bility Hazard Sur- bility Hazard

Time Intrvl Intrvl Risk Events nating viving at End Densty Rate viving Densty Rate

------ ------ ------ ------ ------ ------ ------ ------ ------ ------ ------ ------ ------

,0 404,0 9,0 399,5 4,0 ,0100 ,9900 ,9900 ,0100 ,0101 ,0050 ,0050 ,0050

1,0 391,0 37,0 372,5 6,0 ,0161 ,9839 ,9740 ,0159 ,0162 ,0081 ,0065 ,0066

2,0 348,0 22,0 337,0 2,0 ,0059 ,9941 ,9683 ,0058 ,0060 ,0090 ,0041 ,0042

3,0 324,0 11,0 318,5 1,0 ,0031 ,9969 ,9652 ,0030 ,0031 ,0095 ,0030 ,0031

4,0 312,0 4,0 310,0 4,0 ,0129 ,9871 ,9528 ,0125 ,0130 ,0112 ,0062 ,0065

5,0 304,0 17,0 295,5 2,0 ,0068 ,9932 ,9463 ,0064 ,0068 ,0120 ,0045 ,0048

6,0 285,0 12,0 279,0 3,0 ,0108 ,9892 ,9361 ,0102 ,0108 ,0133 ,0058 ,0062

7,0 270,0 12,0 264,0 1,0 ,0038 ,9962 ,9326 ,0035 ,0038 ,0137 ,0035 ,0038

8,0 257,0 15,0 249,5 3,0 ,0120 ,9880 ,9214 ,0112 ,0121 ,0150 ,0064 ,0070

9,0 239,0 9,0 234,5 1,0 ,0043 ,9957 ,9175 ,0039 ,0043 ,0154 ,0039 ,0043

10,0 229,0 19,0 219,5 ,0 ,0000 1,0000 ,9175 ,0000 ,0000 ,0154 ,0000 ,0000

11,0 210,0 19,0 200,5 4,0 ,0200 ,9800 ,8992 ,0183 ,0202 ,0176 ,0091 ,0101

12,0 187,0 29,0 172,5 2,0 ,0116 ,9884 ,8887 ,0104 ,0117 ,0189 ,0073 ,0082

13,0 156,0 18,0 147,0 1,0 ,0068 ,9932 ,8827 ,0060 ,0068 ,0197 ,0060 ,0068

14,0 137,0 12,0 131,0 3,0 ,0229 ,9771 ,8625 ,0202 ,0232 ,0224 ,0115 ,0134

15,0 122,0 13,0 115,5 ,0 ,0000 1,0000 ,8625 ,0000 ,0000 ,0224 ,0000 ,0000

16,0 109,0 17,0 100,5 1,0 ,0100 ,9900 ,8539 ,0086 ,0100 ,0238 ,0085 ,0100

17,0 91,0 6,0 88,0 ,0 ,0000 1,0000 ,8539 ,0000 ,0000 ,0238 ,0000 ,0000

18,0 85,0 18,0 76,0 2,0 ,0263 ,9737 ,8314 ,0225 ,0267 ,0280 ,0157 ,0189

19,0 65,0 13,0 58,5 ,0 ,0000 1,0000 ,8314 ,0000 ,0000 ,0280 ,0000 ,0000

20,0 52,0 9,0 47,5 ,0 ,0000 1,0000 ,8314 ,0000 ,0000 ,0280 ,0000 ,0000

21,0 43,0 7,0 39,5 ,0 ,0000 1,0000 ,8314 ,0000 ,0000 ,0280 ,0000 ,0000

22,0 36,0 5,0 33,5 ,0 ,0000 1,0000 ,8314 ,0000 ,0000 ,0280 ,0000 ,0000

23,0 31,0 12,0 25,0 ,0 ,0000 1,0000 ,8314 ,0000 ,0000 ,0280 ,0000 ,0000

24,0 19,0 9,0 14,5 ,0 ,0000 1,0000 ,8314 ,0000 ,0000 ,0280 ,0000 ,0000

25,0 10,0 5,0 7,5 ,0 ,0000 1,0000 ,8314 ,0000 ,0000 ,0280 ,0000 ,0000

26,0+ 5,0 5,0 2,5 ,0 ,0000 1,0000 ,8314 ** ** ,0280 ** **

** These calculations for the last interval are meaningless.

This subfile contains: 404 observations

The median survival time for these data is 26,00+
